# The Effect of the Addition of Coal Fly Ash (CFA) on the Control of Water Movement within the Structure of the Concrete

**DOI:** 10.3390/ma16155218

**Published:** 2023-07-25

**Authors:** Grzegorz Ludwik Golewski

**Affiliations:** Department of Structural Engineering, Faculty of Civil Engineering and Architecture, Lublin University of Technology, Nadbystrzycka 40 Str, 20-618 Lublin, Poland; g.golewski@pollub.pl; Tel.: +48-81-5384394; Fax: +48-81-5384390

**Keywords:** coal fly ash, concrete composite, cement matrix, water absorption of concrete, compressive strength, immersion conditions, microstructure, durability

## Abstract

Studies were carried out to find a relation between the important physical property, i.e., water absorption and the main mechanical parameter, i.e., compressive strength (*f*_cm_), of concretes containing coal fly ash (CFA) in the amounts of 0% (CFA-00), 20% (CFA-20%), and 30% (CFA-30). The methodology of the water absorption tests reflected the conditions prevailing in the case of reinforced concrete structures operating below the water table. The microstructure of all materials was also assessed. Based on the conducted studies, it was found that both the *f*_cm_ of concretes with the addition of CFA and its water absorption depended on the percentage of waste used, whereas both analyzed parameters were closely related to the structure of the cement matrix and interfacial transition zone area between the coarse aggregates and the paste. It should be stated that at the content of 20% CFA in the binder composition, an increase in the *f*_cm_ of the material is observed, with a simultaneous increase in its water absorption. On the other hand, the addition of 30% CFA results in a significant decrease in both the strength of the composite and its water absorption. Thus, it was found that in the case of concretes with the addition of CFA, the strength of the material is directly proportional to the level of its water absorption. Moreover, the concrete including 30% CFA may increase the durability of reinforced concrete structures subjected to immersion conditions. From an application point of view, the obtained research results may be helpful in understanding the impact of the CFA additive on the level of water absorption in cement concretes with this waste.

## 1. Introduction

Construction is a fast-growing industrial sector that meets the current trends of industrialization and infrastructure development [1,2,3]. Concrete, on the other hand, is a structural material constituting a module inseparably related to this area of the economy [4,5]. Furthermore, the development of concrete production technology in recent years has gained a pace that has never been recorded in history [6,7].

Unfortunately, this highly useful construction material is still burdened with negative consequences resulting from the use of cement binders for its production [8,9,10]. The basic binder in concrete is ordinary Portland cement (OPC), the production of which is unfortunately the main contributor to greenhouse gas emissions, mainly CO2, associated with global warming and climate change on Earth [11,12,13,14,15].

Therefore, for many years, various types of measures have been taken to reduce the negative effects associated with the production of binders for the production of concrete [16,17,18]. One such undertaking is the substitution of the OPC part in the composition of the concrete mix with other mineral materials [19,20,21,22]. In most cases, they are wastes from various industrial processes [22,23,24,25] and are referred to as Supplementary Cementitious Materials (SCMs) [26,27,28,29,30].

It should also be noted that concretes made with the participation of SCMs allow not only reducing CO2 emissions generated during the burning of the Portland clinker [31] but also significantly reducing the amount of energy necessary to carry out this technological process [32].

The most commonly used SCMs in the cement industry is undoubtedly coal fly ash (CFA) [33,34,35]. In addition, other frequently used SCMs today include:

- Silica fume (SF) [36];
- Ground-granulated blast furnace slag (GGBS) [37];
- Metakaolin (MK) [38];
- Nanosilica (nS) [39];
- C-S-H nanoseeds [40];
- Waste glass, limestone powder, crumb rubber, and others [41].

Coal fly ash is an industrial by-product of hard coal burning in power plants and thermal power plants, and its disposal is a major problem faced by almost all countries in the world [42]. On the other hand, it should be noted that CFA has great potential to be used as a partial replacement for OPC, especially due to its physicochemical properties [43], fine grain size [44], and quite good pozzolanic activity [45]. Therefore, the problem of the effective use of this by-product of coal combustion in cement binder technology and concrete technology has been the subject of numerous studies for many years [46,47,48].

On this basis, it was established that the addition of CFA in the range of up to 20% of cement weight improves the strength of the concrete composite and its fracture toughness both in single and complex stress states [49,50]. It also has a positive effect on reducing the negative effects of vibrations resulting from the occurrence of impact loads and dynamic loads [51,52,53]. This OPC substitute also has high synergistic properties when in contact with other pozzolanic additives [54] and nanoadditives [55].

Moreover, it should be noted that most of the previous research and applications concern mainly concretes with a content of CFA up to 30% by weight of cement, i.e., low-volume fly ash concretes (LVFAC). For this reason, in the presented article, an important physical property affecting the durability of concretes with CFA in relation to LVFAC has been analyzed.

This manuscript presents the assessment of water absorption (WA) for concretes modified with various amounts of CFA additives in the context of the durability assessment of this construction material. In addition to the analysis of the specific property of concrete containing CFA, the presented research also addresses the solution to the above-mentioned problems in terms of reducing CO2 emissions in the cement sector. For this reason, a more in-depth understanding of the next characteristic of CFA concrete may enable an increase in the use of this material in concrete technology and a more sustainable development of the construction industry.

It should be noted that penetration into the concrete structure in an uncontrolled way of both excess moisture and harmful substances, which results in a decrease in its durability and activation of destructive processes in the material much faster [56,57,58]. Furthermore, it should be noted that durability, which in practice means the ability of a building structure to maintain its usable condition, is the most important factor in the field of construction [59]. Moreover, ensuring adequate durability of concrete is crucial to reduce maintenance costs and repairs for reinforced concrete structures involving concretes incorporating SCMs [60].

However, durability in the modern approach is considered in a holistic way, and it depends mainly on the microstructure of concrete [61]. According to [62], durability and strength are mainly controlled by the microstructure of the concrete. A dense microstructure with fewer interconnected pores usually ensures good durability of the concrete [63]. For these reasons, durability evaluation should be based on the analysis of the strength parameters of the concrete composite in conjunction with other important characteristics that have a significant impact on durability, such as the following physical parameters: water absorption [64,65], permeability, or frost resistance [66]. 

Therefore, water absorption of the hardened concrete is one of its basic qualitative features, which is taken into account already at the stage of designing the composition of the concrete mix [67]. In addition, as reported in [68,69,70,71], minimizing water absorption of concrete is important in order to reduce the ingress of chloride-containing or sulfate-containing water into concrete, which can cause serious damage and reduce its durability. Considering the above, it should be stated that a thorough knowledge of the water absorption phenomenon in the case of concretes containing CFA is of decisive importance in the context of forming their durability.

From a physical point of view, water absorption is defined as the amount of water that concrete can absorb at atmospheric pressure. In order to ensure proper durability, its value should be within 4–6% [67], and when the water absorption is below 5%, it is considered to be good quality concrete [72]. According to other data, water absorption of concrete below 10% is considered low [73]. 

From a practical point of view, there is a special group of building structures, which are much more affected by the water absorption problem than other buildings. These structures are primarily hydrotechnical structures, and all other structures occur within their location, e.g., retaining walls.

In addition, one should realize that a significant part of hydrotechnical structures, after their completion and commissioning, works below the water table. Often in such situations, there is also a pressure of water on the structural element. Moreover, the flow of water within such structures is turbulent and thus causes intensive soaking of the concrete and its successive destruction [74].

Therefore, it is very important to use a research methodology based on the assessment of this phenomenon for samples subjected to total immersion in water when assessing the water absorption of concrete. The results of such experiments will reflect, at least partially, the actual working conditions of hydrotechnical structures [75].

Moreover, according to [76], the water absorption test by immersion can be an indirect indication of the pore structure of bulk cement paste in concrete, which has to do with the fact that pores that open to the surface affect water absorption. Thus, the water absorption value is an indirect indication of the porosity of concrete [77]. However, the type of pores (close, open) is also important in this case, which should be carefully assessed.

However, the conditions of testing the water absorption of concrete in the case of soaking samples by contact with water on only one of their surfaces cannot be fully reliable in the assessment of the water absorption of the concretes used in hydrotechnical structures. However, they may concern the assessment of this property in relation to, for example, prefabricated concrete road accessories, e.g., curbs [78].

Water absorption of concrete, as well as its other properties, depends on many material and technological factors. However, its level is determined mainly by the microstructure of the cement paste, including primarily:

- Capillary porosity;
- The distribution of the size of capillary pores;
- The phase composition of the cement matrix.

This, in turn, is due to the fact that the matrix in the concrete composite, as its continuous phase, is the basic trajectory for the migration of water and other substances into the concrete element. The degree of homogenization of the matrix is, in turn, related to:

- The water/cement or water/binder ratio;
- The type of cement;
- The degree of cement hydration;
- The content of mineral additives.

It is well-known that the dense microstructure of the cement matrix, with limited porosity, can be obtained by good compaction and curing of the concrete mix and the use of pozzolanic active mineral additives and admixtures as binder modifiers. Therefore, so far, studies have been conducted to analyze the impact of various modifications on the composition of concrete composites in terms of improving their water absorption. 

On the basis of previous research, it has been found that a significant improvement in the water absorption of concrete is caused by modification of its structure by using:

- 65% GGBFS + 5%SF [62];
- 5% and 10% MK [73,79];
- 5% MK + 0.5% nylon fibers [79];
- 7% and 10% SF [73,80];
- 5% nS [75];
- 20% CFA + 1.5% glass fibers [81].

Previous research on the water absorption of modified concretes also included experiments evaluating this property for composites with the addition of CFA and the implementation of various other types of FA. Therefore, Table 1 summarizes the previous studies on composites with the addition of CFA with the provision of important parameters for these experiments. However, selected data on the implementation of water absorption experiments in concrete with the addition of other types of FA can be found, for example, in the works [82,83,84,85,86,87,88].

At this point, it should also be noted that a beneficial effect on the level of water absorption of the modified concretes, CFA and GGBFS, has been observed in the case of composites exposed to high temperatures [88,89,96,97]. The improvement of this property is also influenced by:

- The replacement of mineral aggregates in the composition of the concrete mix with recycled aggregates [81,89];
- The substitution of sand by FA + GGBFS and GGBFS + SF mixes [98,99];
- A 5% addition of nS and the application of nanopowders to concretes containing CFA [100,101,102].

Analyzing the results of earlier studies on water absorption of concretes—especially those with the addition of CFA in the range of up to 30% of the weight of the cement—it should be stated that the results obtained from various laboratories, using different test methods, do not clearly indicate how the modification of the cement binder composition with this industrial waste affects the water absorption level of concrete composites. Thus, it is also difficult to conclude how such material modification may affect the durability of the concrete in question. It is also ambiguous to associate this physical parameter with the strength of hardened mature concrete. There is also a lack of a thorough analysis and explanation of the results obtained in relation to the microscale of the tested materials. 

In some of the previous studies, concretes with the addition of CFA up to 30%, after 28 days, are characterized by:

- Higher water absorption and, at the same time, reduced compressive strength [86,88];
- Lower water absorption level with obviously still lower compressive strength than in the reference concrete and composites with CFA content below 30% [64,91,92,94].

Since the level of water absorption of concrete is influenced by many factors (as mentioned above), and in addition, there is no clear data on the impact of CFA additive on this important physical property in the scope of concretes evaluated under water immersion conditions (Table 1), extensive experimental research has been carried out in this respect. 

Its goals were the following:

- Evaluation of the influence of the CFA additive in the amounts of 0%, 20%, and 30% on the level of water absorption of concretes and their compressive strength after 28 days of curing;
- Analysis of the microstructure of the cement matrix of the tested concretes;
- Linking the results obtained in the macroscopic tests with observations of the structures of the analyzed composites in order to prepare recommendations helpful in the development of concrete mix compositions with the addition of CFA in an amount of up to 30% with increased durability.

## 2. Materials and Methods

### 2.1. Materials

#### 2.1.1. Aggregates

Locally available natural pit sand with a 2.0 mm maximum size was used as fine aggregate (FA) and natural gravel as a coarse aggregate (CA) with an 8.0 mm maximum size. The properties of both aggregates used were tabulated in Table 2.

#### 2.1.2. Binders

OPC CEM I 32.5 R [103], produced by a local cement plant, and class F CFA [104], produced by a local thermal electric power station, were used as binders in this study. The chemical composition of both binders was evaluated by using the XRF method. The results of these tests were gathered in Table 3.

Moreover, in order to know the mineralogical composition of the binders used, the following measurements methods were introduced:

- Bogue’s method in the case of OPC;
- Rietvield quantitative analysis in the case of CFA.

Based on the above studies, Table 4 and Table 5 contain, respectively, the phase composition of OPC and CFA.

**Figure 1:**
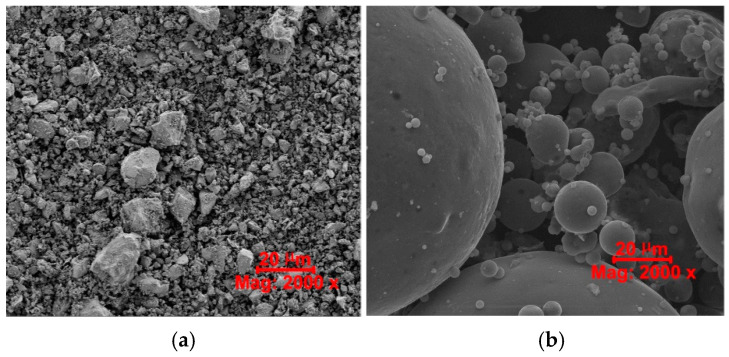
SEM micrographs of the binders used: (**a**) OPC, (**b**) CFA.

In addition, Table 6 contains the physical properties of both fillers, whereas Figure 1 shows photos of the morphology of cementitious materials, i.e., OPC and CFA. Based on these data, it can be concluded that CFA particle size distribution is much finer in comparison to OPC grains.

#### 2.1.3. Water

In these studies, tap water (W) was used, which met the requirements of standard provision EN 1008:2002 [105].

#### 2.1.4. Admixture

A calcium lignosulfonate-based plasticizer (P), Basf Liquol BV-18, used in this study had a density of 1.16 g/cm^3^ and a dosing range of 0.1–1.0% of the mass of cement. The plasticizer was used in an amount of 0.6% of the mass of the binder.

### 2.2. Mix Proportions

Water absorption tests, WA, as well as compressive strength tests, fcm, were conducted on three types of concrete composites containing different amounts of OPC and CFA. Mix proportions were determined based on the following assumptions:

- Constant: the total amount of the binder, the amount of coarse and fine aggregate, water, and the water–binder ratio: w/b = 0.4;
- Different series of concrete containing CFA amounts by weight: 0% (CFA-00), 20% (CFA-20), and 30% (CFA-30).

The mix proportions of the concrete composition are shown in Table 7. 

### 2.3. Mixing, Casting, and Curing of the Specimens

**Figure 2:**
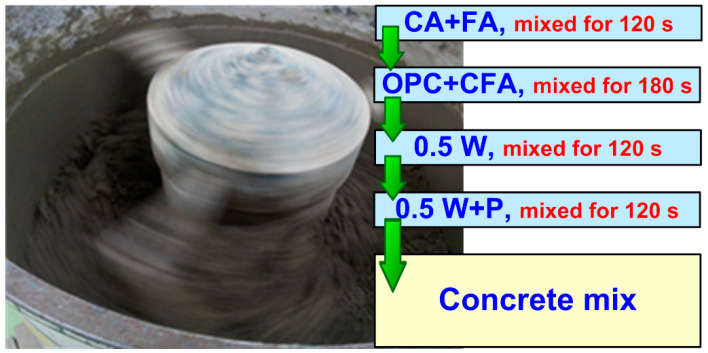
Procedure of mix preparation.

A laboratory concrete mixer with a 100 L capacity was utilized in the preparation of all concrete mixtures. The scheme, including all steps in this procedure, is shown in Figure 2.

Subsequently, the mixture was poured into the molds. Mechanical vibration was applied for casting and compacting the fresh specimens. Cubes with a dimension of 150 × 150 × 150 mm were prepared from each mixture to determine compressive strength parameters and water absorption properties.

After preparing specimens, they were stored in the casting room for 48 h. Specimens were covered by wet burlap to prevent water loss. After demolding the specimens, they were taken to a moist curing chamber and maintained at 20 ± 2 °C and more than 95% relative humidity for the first 14 days. Next, for the following two weeks until testing, the specimens were maintained under laboratory conditions.

### 2.4. Experimental Procedures

#### 2.4.1. Compressive Strength Tests

The compressive strength of concrete, *f*_cm_, was tested in accordance with the European Standard EN 12390-3:2011 + AC:2012 [106] on 150 mm cubes with the use of a Walter +bai ag hydraulic servo testing machine. Six specimens were tested for each concrete type. During the tests, the specimens were loaded statically, the maximum bearing capability was 3000 kN, and the loading rate of the compressive strength test was in a range between 0.5 MPa/s and 0.8 MPa/s.

#### 2.4.2. Determination of Water Absorption

Concrete absorption tests were carried out on cubic specimens with a side length of 150 mm (6 with each series of concrete) and were cured for 28 days by completely immersing the dried cubes (see Section 2). During the studies, the amount of water absorbed per unit of initial mass of the specimens was recorded. 

**Figure 3:**
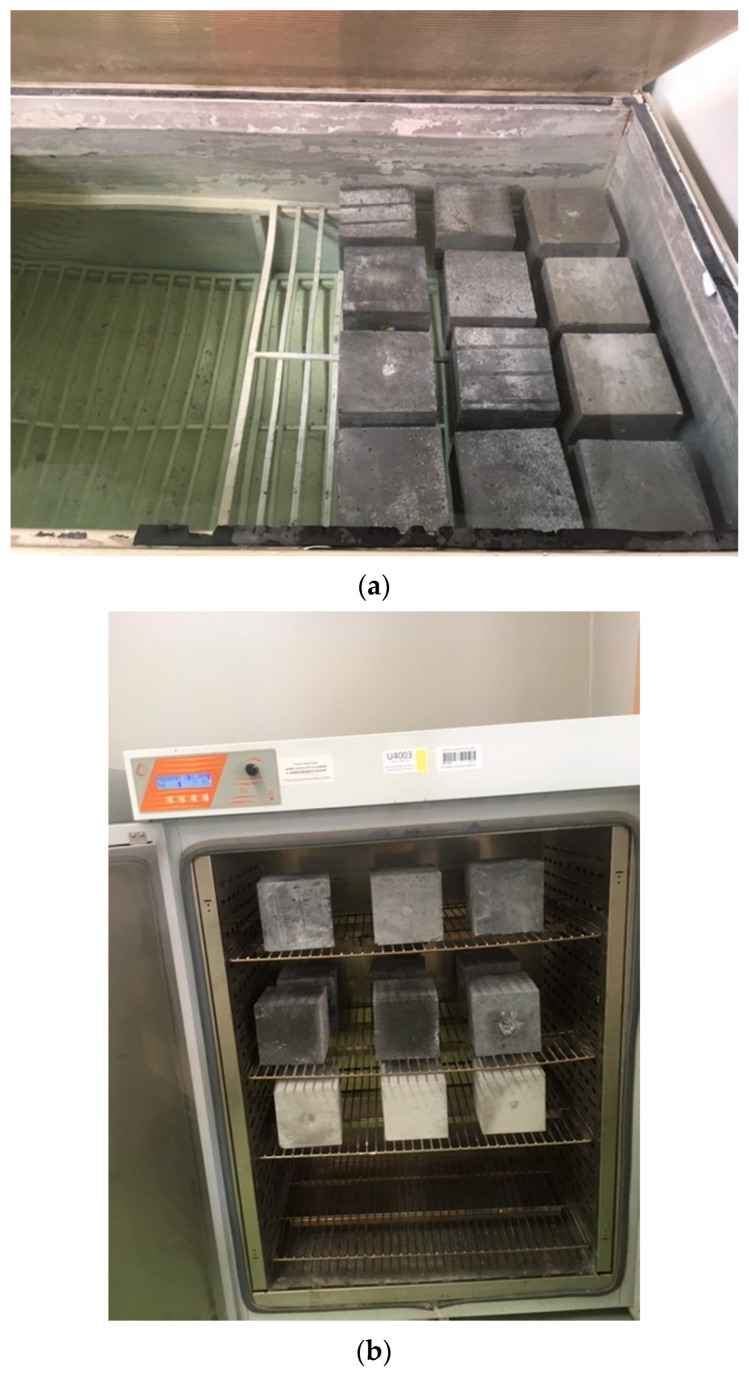
Stages of testing the water absorption of concrete: (**a**) soaking the samples with water in the bath, (**b**) drying the samples to a constant weight in a dryer with hot air circulation.

The complete experiments of measuring the water absorption of the concrete containing CFA consisted of two basic stages shown in Figure 3:

- Soaking the samples with water (Figure 3a);
- Drying them to a constant mass (Figure 3b).

Both in the process of saturating the samples with water and their drying, a number of samples did not exceed twelve pieces during one experiment (Figure 3). However, the detailed course of the procedure related to conducting the experiments was as follows:

- Initially, the samples were placed in a bath vessel on washers with a thickness of about 10 mm so that the distance between the samples was at least 15 mm;
- The bath was filled with water at a temperature of 18 ± 2 °C to half the height of the samples;
- After 24 h, water was added to the bath to a level 10 mm higher than the level of the samples; this level was maintained until the end of sample saturation;
- After another 24 h, the samples were removed from the water, dried to remove excess water, and weighed with an accuracy of 0.2%;
- The saturation of the samples lasted for the following days until two subsequent weighings showed no increase in mass (Figure 3a);
- After the samples were completely saturated, their mass was recorded, and then samples were placed in a dryer with hot air circulation in a Pol-Eko SLW 400 at 105 ± 5 °C (Figure 3b).

The basic essential parameters of the dryer applied are summarized in Table 8.

**Figure 4:**
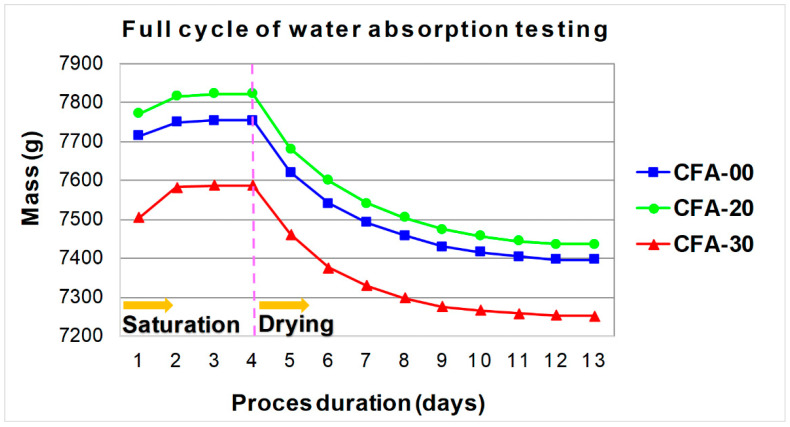
The course of the process of water saturation and drying of the samples.

In the dryer, the samples were dried to a constant weight and the result was recorded again. Measurements of the weight of the samples were made daily, with an accuracy of 1.0 g. Figure 4 shows the full cycle of testing the absorption of water by concrete, divided into the process of saturation and drying of the cubes, with both phases of the experiment marked. It shows that for each of the composites, the entire research process lasted 13 days. 

After this stage, the determination of the water absorption levels in all materials was started. Calculations of water absorption were carried out according to the provisions of two standards:

- PN-88/B-06250 [107], i.e., the “old” Polish standard for ordinary concrete;
- EN 13369 [108], i.e., the standard relating to concrete precast elements.

**Figure 5:**
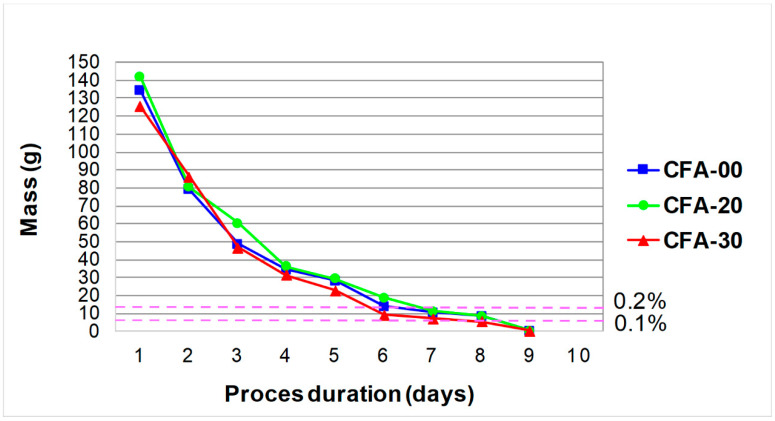
Weight loss of cubes subjected to the drying process with marking the change in the mass of the samples between successive measurements according to both standards.

It should be noted that the water absorption test according to both standards looks the same. The differences in the provisions of both standards consisted in adopting a different percentage level of the difference in the weight of the samples subjected to subsequent drying, after which it was possible to determine the level of their water absorption. In the case of [107], the change in the mass of samples between successive measurements should be below 0.1%, while according to the provisions of the [108] standard, below 0.2%. It is shown in Figure 5.

After determining the moment at which, after two consecutive weighings of the samples, the recommendations of both standards were met, the level of water absorption in the samples was determined. Thus, the water absorption of the concrete specimens was calculated according to Equation (1):

(1) $$\mathrm{WA}=\frac{m_{1}-m_{0}}{m_{0}}\cdot 100\%,$$

where WA is water absorption (%), *m*_1_ is the mass of the sample saturated with water to a constant mass (g), and *m*_2_ is the mass of the sample dried to a constant mass (g).

Figure 5 shows the weight loss of the cubes subjected to the drying process. The graph additionally indicates the time when the change in the weight of samples meets the standard conditions. According to PN-88/B-06250 [107], change in the weight of the sample to below 0.2%, and according to EN 13369 [108], change in the weight of the sample to below 0.1%.

#### 2.4.3. Microstructural Study

A microstructural structure analysis was performed in order to:

- Find the influence of the effect of CFA particles on the water absorption of the analyzed composites;
- Evaluate the air voids of the cement matrixes including CFA;
- Determine the degree of the homogenization of individual materials;
- Assess the degree of reaction of CFA grains;
- Evaluate the Interfacial Transition Zone (ITZ) areas between the coarse aggregate and the paste.

Thanks to this information, it was possible to link the results of the water absorption research with the morphology of the matrixes of individual composites and draw conclusions about their possible susceptibility to penetration of water and other substances into the structure of the analyzed concretes in the context of their durability.

A Quanta FEG 250 Scanning Electron Microscope (SEM) equipped with an energy dispersive spectroscopy (EDS EDAX) was used in this experiment. The procedure and crucial parameters related to the sample preparation process for SEM tests were as follows:

- The shape of the samples—rectangular concrete cube;
- Sample dimensions—10 × 10 × 3 mm;
- Preparation of the samples—taken as raw, i.e., the samples before the test were not polished or prepared in any other way;
- Number of samples—6 for each series of concrete;
- Number of photos per sample—30 photos were taken for each sample, from which the representative photos were selected;
- Magnifications used—200 to 80,000 times;
- Presentations—significant observed details like phases, pores, microcrack width in the ITZ area, and unreacted CFA particles were marked on the selected representative photos.

## 3. Results and Discussion

### 3.1. Mechanical Characterization and the Rate of Water Absorption

**Figure 6:**
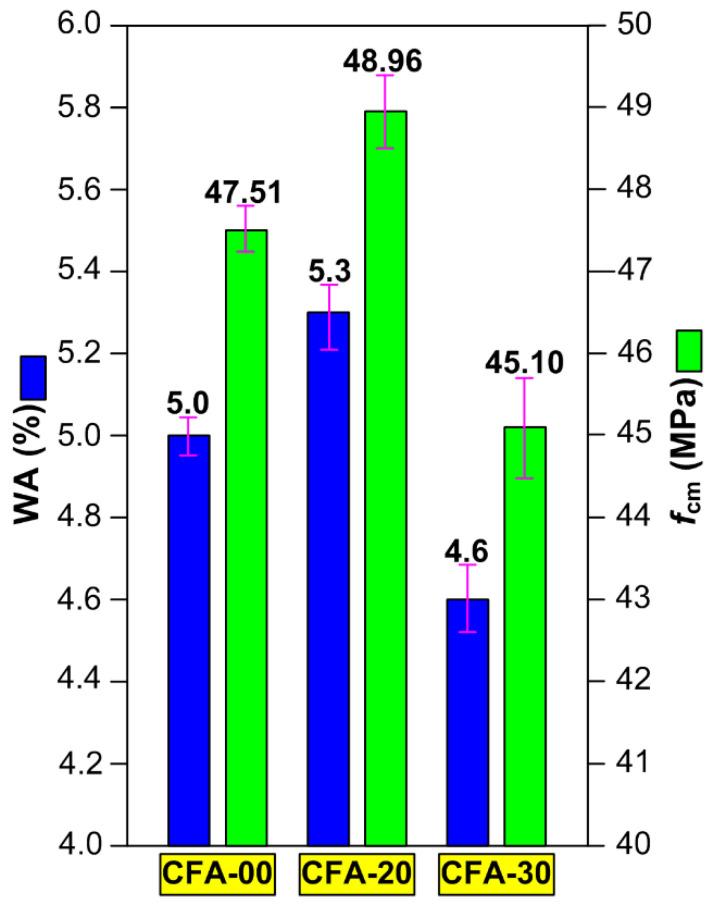
The collective results of the analyzed macroscopic parameters of concrete composites.

Figure 6 shows, collectively, the mean values with error bars of the concrete compressive strength results as well as the water absorption test results at 28 days of age. It can be seen that the results showed low dispersion between them and there was a good correlation between the values. Therefore, the obtained test results can be considered to be fully authoritative.

Based on the results in Figure 6 it can be stated that *f*_cm_ and WA were 47.51 MPa, 5.0%; 48.96 MPa, 5.3%, and 45.10 MPa, 4.6% for test concrete specimens CFA-00, CFA-20, and CFA-30, respectively. Therefore, it can be concluded that:

- The highest compressive strength had concrete CFA-20, while the lowest had CFA-30;
- When the content of CFA increased, the eater absorption of the composites with this waste clearly decreased (Figure 6);
- The compressive strength behaved proportionally to the absorption by immersion (Figure 6), which was also in line with the results of other studies of concretes containing CFA [64,83,85,88].

The obtained results can be explained by the different nature of the phenomena affecting the destructive processes in concrete, which differs from the factors determining the level of its permeability. This is especially true for concrete with the addition of CFA because in such materials CFA grains clearly modify both the ITZ area and the basic structure of the cement paste [109].

It should be noted that the mechanism of transporting media and moisture deep into the concrete structure is mainly based on its migration through the system of large capillary pores in the area of the continuous phase of concrete, which is the cement matrix. However, the mechanism of concrete destruction is different and based on the development and propagation of microcracks through the contact zones of aggregate grains with cement paste.

Although the ITZ area occupies as much as 1/3 to 1/2 of the entire volume of the hardened cement paste in concrete, it is known that it has a microstructure different from the microstructure of the paste inside the concrete composite. Since it is also the place where the first microcracks in concrete occur, it would seem that it may also have a significant share in the permeability of concrete, and thus affect the level of its water absorption. However, Ref. [110] shows that despite the high porosity of ITZ, the permeability of concrete is determined by the basic mass of the hardened cement paste, which is the only continuous phase in concrete. This is also confirmed by the fact that the permeability of the hardened cement paste is lower than the concrete made on the basis of this paste.

**Figure 7:**
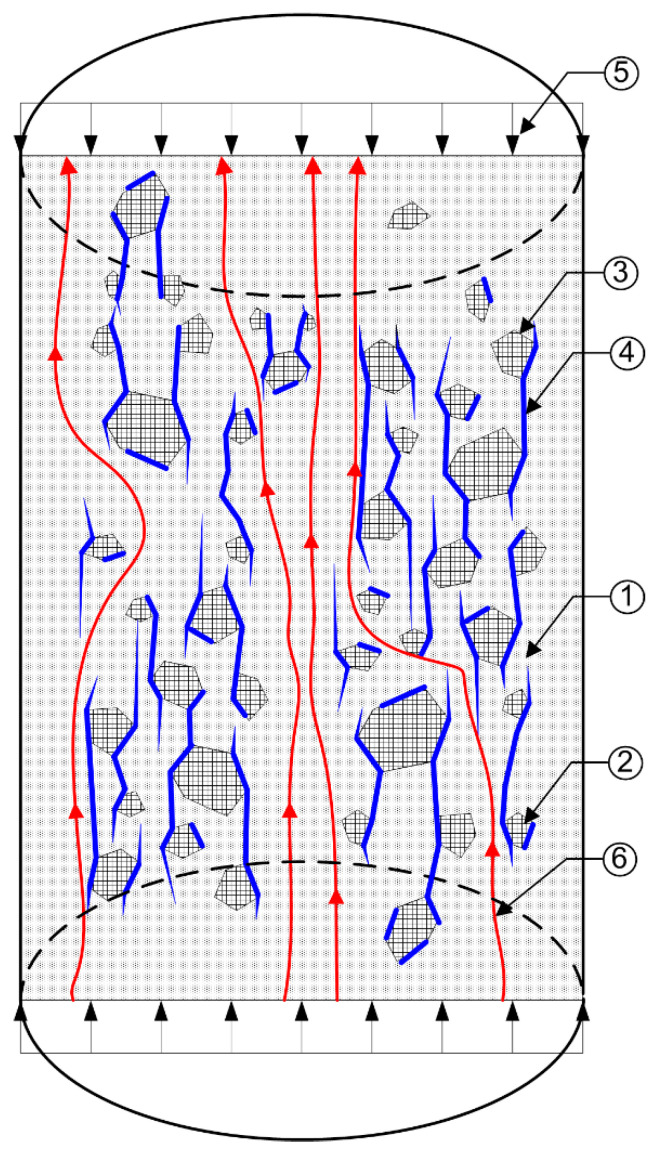
Diagram illustrating the mechanism of concrete destruction due to the development of microcracks in ITZ areas and places of media penetration through the continuous phase of concrete: 1—cement matrix, 2—aggrgate grain, 3—ITZ, 4—microcrack, 5—load, 6—the path of media penetration through the continuous phase of concrete; the description is in the text.

The differences in both physical phenomena are shown in the example of the structure of the loaded concrete sample in Figure 7.

Figure 7 shows the mechanism of the destruction of concrete material, which is directly affected by ITZ between the coarse aggregate grains and the paste. The quality of ITZ determines mainly the strength of concrete stone [111]. It is in these zones that the first microcracks in the composite are initiated. Then, as the load increases, the microdamages propagate and accumulate, which in turn leads to the destruction of the material (Figure 7). Therefore, a more homogeneous and compact ITZ implies both an increase in concrete strength and a delay in the process of its destruction [112]. Microcracks and the paths of their propagation are marked in blue in Figure 7. 

In turn, water absorption is directly related to the structure of the continuous phase of concrete, i.e., the cement matrix. The greater tightness of this phase of the composite reduces the level of material water absorption. As mentioned above and demonstrated in [110], the ITZ area has a smaller impact on the level of concrete water absorption, which is primarily determined by the level of porosity and type of pores in the cement paste structure. In this zone, there is the penetration of moisture and harmful substances that largely reduces the durability of the composite. The paths of media penetration through the concrete structure are marked with red arrows in Figure 7. 

Such a phenomenon has been observed among others in the case of composites with the addition of CFA, which do not contain coarse aggregate grains in their composition, i.e., mortars. In these composites, due to the limitation or even complete absence of weak points in the form of ITZ in the area of large aggregate grains, the strength of such materials shows convergence with the levels of their water absorption [105]. A similar trend can also be observed for composites with a high CFA content, i.e., high-volume fly ash concrete (HVFAC) [89,101]. 

Therefore, in order to determine the exact mechanism affecting the obtained test results, including primarily the water absorption, microstructural analyses of pieces of cement matrixes separated from the structures of all concretes have been carried out. In addition, the ITZ areas of all composites have been also studied. Association of observations of the microstructures of both zones of the composite—determining both the strength of the concrete and the level of its water absorption—was supposed to give an answer to the question of how the modification of the cement binder composition by the addition of CFA affects the change in the analyzed parameters in the concrete in question.

### 3.2. SEM Analysis

**Figure 8:**
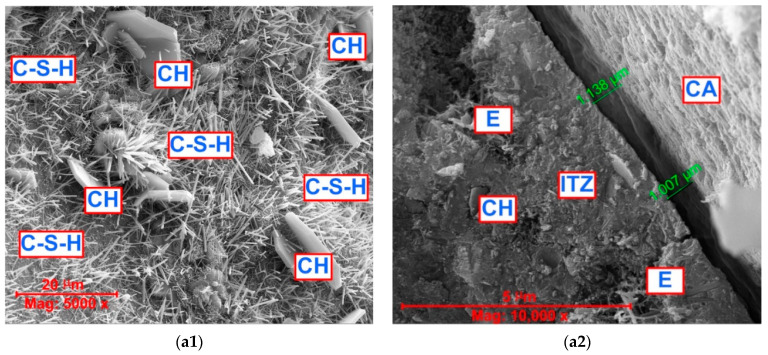

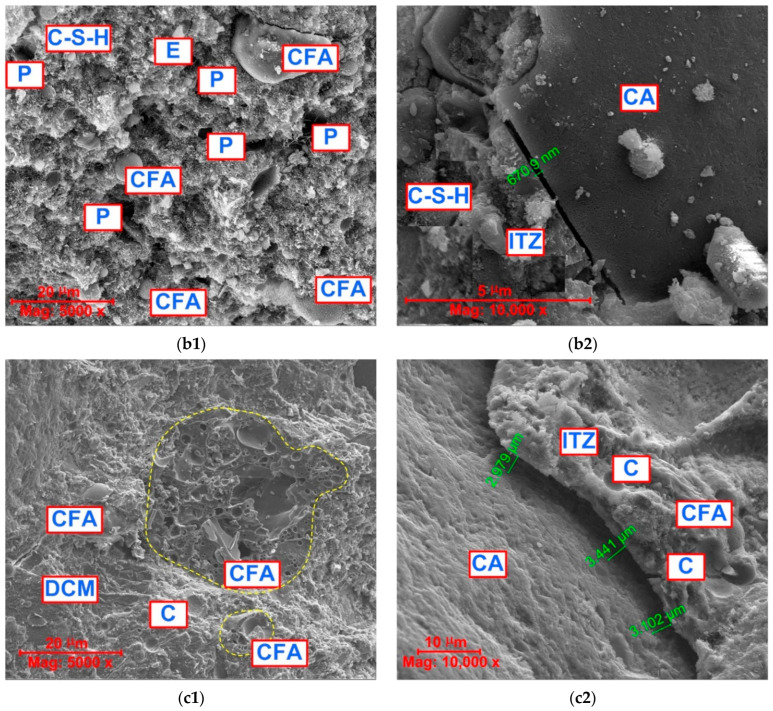
Typical microscopic photos of the matrix and ITZ in concretes with CFA: (**a1**,**a2**) CFA-00, (**b1**,**b2**) CFA-20, (**c1**,**c2**) CFA-30; CFA—coal fly ash, P—pore, C—cavitie created after separating the CFA grain, E—ettringite, CH—Portlandite, C–S–H—calcium silicate hydrate, CA—coarse aggregate, ITZ—Interfacial Transition Zone, DCM—dense cement matrix; description in the text.

The SEM photos for the three types of analyzed concrete samples with the same magnification (5000 and 10,000 times) are shown in Figure 8. For each of the concretes, two representative photos are shown. They show both the structure of the cement matrix (photos marked as 1) and the ITZ areas (photos marked as 2).

Based on the SEM studies, it can be stated that the cement matrix of reference concrete, i.e., CFA-00 (Figure 8(a1)):

- Presented the typical microstructure of unmodified concrete and contained mainly the continuously developing phases of calcium silicate hydrate (C-S-H) and calcium hydroxide (CH);
- Contained both the fibrous phase of the C-S-H and C-S-H structures that look like honeycombs [113] (Figure 8(a1)).

The implementation of the CFA additive into the binder composition caused, in addition to products related to the hydration reaction, additional phases in the cement matrix structure resulting from the pozzolanic reaction to appear. As shown by the detailed results of the CFA pozzolanic activity studies, in the case of substitution of 25% OPC with this waste, a significant positive effect of strengthening the matrix based on a binary binder is observed [109]. In the presented studies, OPC substitution was used at levels of 20 and 30%. 

Analyzing the appearance of the structure of the cement matrix shown in Figure 8(b1), i.e., for CFA-20 series concrete, it should be stated that the 20% amount of CFA was probably too small to significantly strengthen the structure of the composite after 28 days of curing. Unfortunately, this caused the concrete of this series to be (Figure 8(b1)):

- Clearly porous;
- Have loose clusters of the C-S-H phase;
- Include a few unreacted CFA grains.

For the above reasons, this composite is characterized by the highest water absorption by immersion (Figure 6). 

An increase in the amount of CFA in the composition of the binder by 10% resulted in a clear intensification of the pozzolanic reaction processes in concrete after 28 days of its curing. Thanks to this, the structure of the cement matrix in this concrete was (Figure 8(c1)):

- Compact and dense;
- The unhydrated CFA particles and cavities created after separating the CFA grains still existed.

Nevertheless, in this concrete, the reacting CFA grains were clearly visible (the area marked with a dashed yellow line in Figure 8(c1)), which had a clear impact on the sealing of the matrix structure and, consequently, the reduction in the water absorption level in the concrete in the CFA-30 series (Figure 6).

It should be noted that the results of the microstructural tests presented above confirm the results of other experiments evaluating the water absorption of concretes with the addition of CFA, for example:

- Increasing the CFA content decreased the sorptivity and capillary absorption of the concrete significantly [76,114,115];
- The rate of water absorption decreased with an increase in CFA level due to the reduction in the pore size with the incorporation of CFA [64];
- At low water-to-binder ratios, CFA leads to significantly smaller capillary absorption rates regardless of curing age [116];
- The pozzolanic reaction of CFA progresses significantly and consumes large amounts of CH, producing additional secondary C-S-H gels and resulting in denser and more compact microstructures, thus decreasing the amount of water absorption by immersion [82].

During the detailed analysis of the microstructure of all three composites, the ITZ areas in the places at the largest inclusions have been also assessed. Both the morphology of this zone and the size of the microcracks occurring at the border of the two main phases of concrete have been assessed. As previous studies have shown, the width of the microcracks in the ITZ area is directly related to the strength of the composite and its fracture toughness [117].

As a result of this analysis, it has been determined that the structure of the reference concrete contains numerous phases in the ITZ area in the form of ettringite and portlandite. Microcracks in the ITZ area have a size of approx. 1 μm (Figure 8(a2)). 

In turn, the smallest sizes of microcracks in the ITZ area, significantly below 1μm, have been recorded in the CFA-20 series concrete. The ITZ area in this concrete has been compact, with a fairly ordered structure (Figure 8(b2)). 

The most heterogeneous structure has been observed in CFA-30 series concrete. In the ITZ area of this material, unreacted CFA grains and places after their separation from the matrix structure have been visible. In this concrete, cracks with the largest opening widths have been also visible (Figure 8(c2)). 

On the basis of the comprehensive microstructural analyses related to the assessment of significant parameters of the analyzed concretes and the available literature data, it can be concluded that:

- Both the compressive strength of the concrete with the addition of CFA and its water absorption depend on the percentage of waste used;
- Both analyzed parameters are closely related to the structure of the cement matrix and ITZ areas in between the coarse aggregate grains and the paste.

It has been observed that the addition of CFA in the range of up to 20% is able to positively change the ITZ microstructure. This zone is quite compact, and the microcracks are very small (Figure 8(b2)). This means that the strength of the concrete with the addition of 20% CFA is the highest. However, observing the paste structure in this material, it should be stated that it is strongly porous and heterogeneous. Therefore, it seems that the 20% addition of CFA is definitely sufficient to change the structure of ITZ, but it is not enough to comprehensively strengthen the structure of the cement matrix. Moreover, this amount is also too small to change the size and structure of the pores of the concrete composite. In previous studies, it has been observed that the highest activity had OPC-CFA mixtures with a CFA content of 25% [109]. This work [109] also presented chemical details related to the formation of a compact matrix structure with CFA from the first hours of its curing to a year.

The content of CFA above 20% causes the sealing of the cement matrix structure by [118]:

- Reducing the content of large pores;
- Changing in the structure of the pores, i.e., a reduction in the share of large open capillary pores and the appearance of closed micropores of very small size;
- Reducing the CH content in the paste;
- Increasing the C-S-H phase content.

However, excessive CFA content is not beneficial from the point of view of increasing concrete strength [119]. Previously, it has been shown that the boundary content of CFA in concrete is approx. 30% [120]. In this case, the excess CFA is no longer able to fully react. Therefore, unreacted grains and voids appear in the structure—both the cement matrix and ITZ—after their separation from the paste structure (Figure 8(c2)). However, in relation to the cement matrix, this does not create a problem because it is already sufficiently compacted. Voids after CFA grain separation, which may weaken the properties of ITZ and affect the size of microcracks in this area. For this reason, concrete with 30% CFA addition has achieved the lowest compressive strength. Simultaneously, this material was the tightest and was characterized by the lowest level of water absorption (Figure 8(c1)).

Therefore, it can be concluded that in the case of concretes with the addition of CFA, the strength of the material is directly proportional to the level of its water absorption. So, it is a completely different phenomenon from that observed in the case of ordinary concrete. Based on the conducted research, it has been observed that (Figure 6):

- At a content of 20% CFA in the binder composition, an increase in the compressive strength of the material is observed, with a simultaneous increase in its water absorption;
- Addition of 30% CFA results in a significant decrease in both the strength of the composite and its water absorption.

It should be added that a similar phenomenon of a decrease in the level of water absorption in low-volume fly ash concrete has been also observed in research by other authors. According to [64], the level of water absorption decreases in the case of cement binder substitution by CFA in a range of up to 35%. However, studies [83,84,85] prove that the addition of CFA from 30 to 90% causes a decrease in water absorption in these materials. However, the low water absorption in these studies was observed in concrete with 30% CFA.

From an application point of view, the obtained research results may be helpful in understanding the impact of the CFA additive on the level of water absorption in cement concretes with this mineral additive. As a consequence, such knowledge can be used for a more conscious designing of concrete structures made of such material, which during their operation would be exposed to total immersion in water.

## 4. Conclusions

The idea behind this research was to produce concrete with improved durability and reduced CO_2_ impact with regard to its use in reinforced concrete structures subjected to total immersion during operation. This was achieved by using partial OPC replacement with CFA. The compressive strength, water absorption, and microstructure at the age of 28 days were investigated. Based on the results obtained during this research, the following conclusions can be made:

(1) Compressive strength increased in concrete containing 20% CFA, i.e., in CFA-20, and decreased with an increase in CFA content up to 30%, i.e., in CFA-30;
(2) Coal fly ash concrete made with up to a 30% replacement level showed better absorption behavior than reference concrete;
(3) Both the compressive strength of concrete with the addition of CFA and its water absorption depend on the percentage of waste used, whereas both analyzed parameters are closely related to the structure of the cement matrix and ITZ areas between the coarse aggregate grains and paste;
(4) At a content of 20% CFA in the binder composition, an increase in the compressive strength of the material is observed, with a simultaneous increase in its water absorption. On the other hand, the addition of 30% CFA results in a significant decrease in both the strength of the composite and its water absorption. Therefore, the compressive strength in concretes containing CFA behaved proportionally to the absorption by immersion;
(5) The hydration products of concrete mixed with 20% CFA showed a loose and porous microstructure, and its water absorption increased accordingly;
(6) The concrete composites including 30% CFA may increase the durability of reinforced concrete structures subjected to immersion conditions;
(7) Finally, more investigations are needed on other parameters such as porosity, permeability, and depth of water penetration aspects of concrete made with CFA in order to better understand the influence of this waste on these specific and significant properties of concrete affecting the durability of these composites. The results of these studies will be the subject of subsequent publications.

## Figures and Tables

**Table 1:** Papers including the results of water absorption of concretes containing CFA.

Addition of CFA (%)	Age of Samples	Results and the Main Conclusions from the Studies	Reference
15; 25; 35	28	WA decreased with an increase in the amount of CFA	[64]
25	28; 90	WA ranged from 4.8% after 28 days to 5.1% after 90 days	[82]
40; 55; 70	28; 90	Larger quantities of CFA lead to higher WA after 28 days	[89]
		After 90 days of curing, the amount of WA decreased	
30; 50; 70; 90	28	WA ranged from 4.4 to 7.3% The low WA was observed in concrete with 30% CFA Other concretes showed higher WA than the control concrete	[90]
30; 60	28	WA decreased with an increase in the amount of CFA	[91]
30; 50	28	Larger quantities of CFA lead to lower WA	[92]
7.5; 15; 25	28	WA increased with an increase in the amount of CFA	[93]
15	28	The addition of CFA decreased WA	[94]
22	90	WA ranged from 4.3 to 8.0%	[95]

**Table 2:** Properties of the fine and coarse aggregates.

Property	Unit	Aggregate Type	
		Fine Aggregate (FA)	Coarse Aggregate (CA)
Specific density	(g/cm^3^)	2.60	2.65
Bulk density	(g/cm^3^)	2.20	2.25
Compressive strength	(MPa)	33	34
Modulus of elasticity	(10^2^ MPa)	330	330
Sand point for the aggregate mix	(%)	40.7	

**Table 3:** Chemical composition of the binders used (mass %).

Material\Constituent	SiO_2_	Al_2_O_3_	CaO	MgO	SO_3_	Fe_2_O_3_	K_2_O	P_2_O_5_	TiO_2_	Ag_2_O
**OPC**	15.00	2.78	71.06	1.38	4.56	2.72	1.21	-	-	-
**CFA**	55.27	26.72	2.35	0.81	0.47	6.66	3.01	1.92	1.89	0.10

**Table 4:** Mineralogical composition of OPC (mass %).

Phase Type	C3S	C2S	C3A	C4AF
Contents	60.69	15.82	9.24	7.28

**Table 5:** Phase composition of CFA (mass %).

Phase Type	Vitreous	Crystalline	
		Quartz (SiO_2_)	Mullite (Al_6_Si_2_O_13_)
Contents	71.5	19.7	8.8

**Table 6:** Physical properties of the binders used.

Type	Analyzed Parameter			
	Specific Gravity (g/cm^3^)	Specific Surface Area (m^2^/g)	Average Particle Diameter (μm)	Color (Visually)
OPC	3.11	0.33	40.0	Light gray
CFA	2.14	0.36	30.0	Dark gray

**Table 7:** Mix proportioning (kg/m^3^).

Mix	OPC	%OPC	CFA	%CFA	W	P	FA	CA
CFA-00	352	100	0	0	141	2	676	1205
CFA-20	282	80	70	20	141	2	676	1205
CFA-30	246	70	106	30	141	2	676	1205

**Table 8:** Parameters of the Pol-Eko SLW 400 dryer used for drying the samples during the WA test.

Parameter	Date
Air circulation	Natural
Chamber volume (L)	400
Chamber material	Acid-proof stainless steel
Outline dimensions (mm) width × height depth	1240 × 1140 × 800 + 60 (handle)
Chamber dimensions (mm) width × height × depth	995 × 790 × 510
Operating temperature range (°C)	5 °C above ambient temperature up to 250 °C
Rated power (kW)	3500
Temperature regulation (°C)	co 0.1 °C
Temperature stability at 105 °C (°C)	±0.5 °C
Number of shelves standard/max	3/11

## Data Availability

No new data were created or analyzed in this study. Data sharing is not applicable to this article.
